# Documented Obstetric Haemorrhage in a General Intensive Care Unit in Ghana: Outcomes and the Unadjusted Association Between Recorded GCS and Mortality

**DOI:** 10.3390/jcm15218228

**Published:** 2026-10-22

**Authors:** Rex Mawuli Kwadjo Djokoto, Irene Bandoh, Wilfred Kwamina Junior Sam-Awortwi, Nana Addo Boateng, William Addison, Moses Siaw Frimpong, Edward Anabila Agana, Kingsley Afreh Nduroh, Johnny Arthur-Komeh, Opei Adarkwa, Andrew Panyin Vormawor, Amponsah Peprah, Anthony Amanfo Ofori, Augustine Tawiah

**Affiliations:** 1 Department of Obstetrics and Gynaecology, School of Medical Sciences, Kwame Nkrumah University of Science and Technology, Kumasi P.O. Box 1984, Ghanaofooh@yahoo.com (A.A.O.);; 2 Department of Obstetrics and Gynaecology, Oak Specialist Hospital, KNUST, Kumasi P.O. Box 420, Ghana; 3 Directorate of Anaesthesia and Intensive Care, Komfo Anokye Teaching Hospital, Adum, Kumasi P.O. Box 1934, Ghana; bandohirene@gmail.com (I.B.); boatengnana@yahoo.com (N.A.B.);; 4 Department of Anaesthesiology, School of Medical Sciences, Kwame Nkrumah University of Science and Technology, Kumasi P.O. Box 1984, Ghana; 5 Department of Obstetrics and Gynaecology, Komfo Anokye Teaching Hospital, Adum, Kumasi P.O. Box 1934, Ghana; 6 Department of Obstetrics and Gynaecology, Powerhouse Hospital, Old Tafo, Kumasi P.O. Box SE 1573, Ghana; 7 Department of Obstetrics and Gynaecology, Manhyia Government Hospital, Kumasi P.O. Box 1908, Ghana

**Keywords:** obstetric haemorrhage, postpartum haemorrhage, maternal mortality, critical care, intensive care, Ghana

## Abstract

**Background:** Obstetric haemorrhage remains an important cause of maternal death and severe morbidity. Outcomes depend on timely recognition, resuscitation, referral, access to blood and essential treatment, and critical care. This study described the clinical characteristics, documented haemorrhage diagnoses, and ICU outcomes of women admitted with obstetric haemorrhage to the main intensive care unit (ICU) of Komfo Anokye Teaching Hospital (KATH), Kumasi, Ghana. **Methods:** This retrospective cohort analysis included 79 women with an obstetric haemorrhage-related diagnosis documented in ICU records, drawn from 344 obstetric ICU admissions between 17 July 2022 and 16 July 2023. Demographic and clinical characteristics, ICU treatments, and outcomes were summarized. Survivors and non-survivors were compared using the Wilcoxon rank-sum test and the chi-square or Fisher exact test, as appropriate. Only unadjusted comparisons are reported because the patient-level dataset required for a prespecified admission-only model was not available for analysis. **Results:** Among 344 obstetric admissions to KATH’s main ICU, 79 women (23.0%) had documented obstetric haemorrhage and were included. Postpartum haemorrhage was the most common diagnosis (32/79, 40.5%), followed by abruptio placentae (18/79, 22.8%) and uterine rupture (12/79, 15.2%). Forty-four women died in the ICU (55.7%). The GCS variable, labelled “admission GCS” in the source dataset, was lower among non-survivors than survivors (median 8 vs. 15; *p* < 0.001), as was diastolic blood pressure (71.5 vs. 86 mmHg; *p* = 0.010). Mechanical ventilation was more frequent among non-survivors (70.5% vs. 40.0%; *p* = 0.013), while hospital stay was shorter (4 vs. 9 days; *p* < 0.001). GCS measurement timing could not be verified; treatment and length-of-stay differences describe post-admission care and course. **Conclusions:** Among women admitted to a general mixed ICU with documented obstetric haemorrhage, ICU mortality was high and postpartum haemorrhage was the most common diagnosis. Lower recorded GCS among non-survivors was an unadjusted descriptive finding; the source dataset’s GCS timing could not be confirmed, and no adjusted prognostic effect can be inferred. Prospective studies with verified admission measures, explicit case criteria, and treatment timing are needed.

## 1. Introduction

Maternal mortality remains a major public health challenge worldwide despite substantial progress in maternal healthcare. The World Health Organization estimated that approximately 260,000 women died during and following pregnancy and childbirth in 2023; about 92% of maternal deaths occurred in low- and lower-middle-income countries, and sub-Saharan Africa accounted for approximately 70% [1]. Many maternal deaths are preventable through timely and effective care; however, prevention depends on reliable systems for recognition, referral and transport, blood and essential treatment, and critical care. In low-resource settings, gaps across these services can delay or prevent life-saving care [1,2].

Obstetric haemorrhage remains one of the leading direct causes of maternal mortality worldwide. It is estimated to account for approximately one-quarter of all maternal deaths globally and continues to be a major contributor to severe maternal morbidity [3,4]. Obstetric haemorrhage encompasses conditions including postpartum haemorrhage, antepartum haemorrhage, placental abruption, placenta previa, uterine rupture, and haemorrhagic shock [5]. Among these conditions, postpartum haemorrhage is responsible for the majority of haemorrhage-related maternal deaths worldwide [3,6]. Major obstetric bleeding can rapidly progress to hypovolaemic shock, disseminated intravascular coagulation, multi-organ failure, and death if prompt treatment is unavailable [5,6].

In Ghana, maternal mortality remains an important public health concern. National and hospital-based studies have consistently identified obstetric haemorrhage as one of the leading causes of maternal death [7,8,9]. Earlier studies reported haemorrhage as the principal cause of maternal mortality [8,9], while more recent evidence suggests that hypertensive disorders of pregnancy have overtaken haemorrhage as the leading cause in some settings [7,10]. Nevertheless, obstetric haemorrhage continues to account for a substantial proportion of maternal deaths across the country [9,11]. Postpartum haemorrhage remains a significant contributor to maternal mortality in urban tertiary facilities [10], and case-level evidence from rural Ghana illustrates how delays in referral and gaps in blood and oxygen availability can turn postpartum haemorrhage into a fatal event outside specialist centres [12].

Several studies in Ghana have examined maternal mortality trends, causes of maternal death, and health-system factors contributing to poor outcomes [7,8,9]. A ten-year review from Cape Coast Teaching Hospital included 64 women admitted to an obstetric ICU with haemorrhage and reported a case-fatality rate of 37.5% [13]. A hospital-based KATH review reported a 5.9% case-fatality rate among haemorrhage admissions overall, a broader population not restricted to ICU patients [7]. These studies show that Ghanaian evidence on haemorrhage-related mortality already exists, while estimates from different care settings should not be compared as if they were equivalent. The present diagnosis-specific analysis describes women with documented obstetric haemorrhage managed in KATH’s main mixed general ICU. It focuses on 79 women drawn from the 344-admission parent cohort and does not claim to be the first Ghanaian study of haemorrhage-related ICU outcomes. Because critically ill women may arrive after substantial physiological deterioration, describing their presentation and outcomes can inform future risk assessment and referral planning.

This study aimed to describe the clinical characteristics, documented haemorrhage diagnoses, and outcomes of women admitted with obstetric haemorrhage to the main ICU of Komfo Anokye Teaching Hospital (KATH), Kumasi, Ghana, and to compare unadjusted characteristics between ICU survivors and non-survivors.

## 2. Methodology

### 2.1. Study Design

This retrospective cohort analysis included women with a documented obstetric haemorrhage-related diagnosis who were admitted to the main ICU of Komfo Anokye Teaching Hospital (KATH), Kumasi, Ghana. The analysis describes clinical characteristics, haemorrhage diagnoses, ICU treatments, and outcomes, and presents unadjusted comparisons by ICU survival status.

### 2.2. Study Setting

The study was conducted at the main intensive care unit of Komfo Anokye Teaching Hospital (KATH), Kumasi, Ghana. KATH is a large tertiary referral hospital serving patients from across Ghana. Women in this cohort were managed in the main mixed general ICU, which admitted obstetric and non-obstetric patients; the findings do not describe care in KATH’s more recently established dedicated maternal ICU. The main ICU provides advanced critical care for women with severe obstetric and gynaecological conditions requiring intensive monitoring and organ support. It receives referrals from maternity, district, regional, and other tertiary hospitals. Multidisciplinary management is provided by obstetricians, anaesthesiologists, intensivists, nurses, and allied health professionals.

### 2.3. Study Population

The study included women admitted to KATH’s main ICU during the parent-cohort period, 17 July 2022 to 16 July 2023, whose primary or final diagnosis in the patient record documented obstetric haemorrhage or a related condition. Eligible diagnoses included postpartum haemorrhage, antepartum haemorrhage, placental abruption, placenta previa, uterine rupture, haemorrhagic shock, and other haemorrhage-related obstetric diagnoses. The 79 cases were selected from the admissions list for the whole period; however, the ICU admission register was unavailable, so the exact dates of the first and last haemorrhage-specific admissions could not be independently confirmed. Each woman contributed one qualifying admission, and no patient had more than one qualifying episode. We could not verify a separate KATH operational definition of “severe obstetric haemorrhage” applicable to these records. Inclusion was based on the documented clinical diagnosis and ICU admission, not on a uniform objective threshold such as estimated blood loss, transfusion volume, or haemodynamic criteria. Accordingly, we refer to the cases as documented obstetric haemorrhage and do not imply a standardized measured severity category.

The source cohort comprised 344 obstetric ICU admissions and has also been analysed in a companion manuscript covering all obstetric ICU admissions and in-hospital mortality [14]. The present paper focuses exclusively on the 79 women with documented obstetric haemorrhage and their diagnosis-specific characteristics and ICU outcomes. Because both papers draw on the same parent cohort, some patient records and descriptive cohort information may overlap; this report is a haemorrhage-specific analysis, not an independent cohort.

### 2.4. Data Collection

Data were extracted retrospectively from the hospital ICU database and patient medical records using a standardized data collection form. Information included demographic characteristics, obstetric history, admission diagnoses, clinical parameters, ICU treatments, and outcomes. Collected variables included age, parity, Glasgow Coma Scale (GCS) score, heart rate, systolic and diastolic blood pressure, oxygen saturation, respiratory rate, hospital length of stay, mechanical ventilation, vasopressor support, central venous catheterization, continuous positive airway pressure (CPAP), haemodialysis, tracheostomy, supplemental oxygen, and ICU outcome. The aggregate dataset labels the GCS variable as “admission GCS,” but the available materials did not establish whether this was measured at ICU arrival or was the first GCS recorded during the ICU stay; timing relative to sedation or intubation was also unavailable. ICU treatment variables refer to care recorded during the admission and were not treated as baseline characteristics. Blinding of data abstractors and inter-rater reliability testing were not documented.

### 2.5. Outcome Measure

The primary outcome was ICU mortality, defined as death occurring during admission to KATH’s main ICU. Patients were classified as survivors or non-survivors according to their ICU disposition. ICU mortality is used consistently as the primary endpoint throughout this manuscript.

### 2.6. Statistical Analysis

Data were analyzed using R statistical software, version 4.6.1 (R Foundation for Statistical Computing, Vienna, Austria). Continuous variables were assessed for distribution and summarized as medians and interquartile ranges (IQRs). Categorical variables were summarized as frequencies and percentages. Survivors and non-survivors were compared using the Wilcoxon rank-sum test for continuous variables and the chi-square or Fisher exact test for categorical variables, as appropriate. Statistical significance was defined as a two-sided *p*-value < 0.05. Only unadjusted comparisons are reported. The patient-level dataset needed to fit a prespecified admission-only model was unavailable for analysis; therefore, we report no multivariable model, Cox sensitivity analysis, C-statistic, or calibration analysis, and infer no independent predictors.

### 2.7. Ethical Considerations

The parent protocol, Implementing Triage and Quality Improvement Interventions to Improve Outcomes for Obstetric Emergency Care, was approved by the Komfo Anokye Teaching Hospital Institutional Review Board (KATH IRB; reference KATH IRB/AP/108/23) on 4 July 2023 and remained valid through 3 July 2024. Patient records were anonymised before analysis, and confidentiality was maintained.

## 3. Results

### 3.1. Cohort Characteristics

A total of 344 obstetric patients were admitted to KATH’s main ICU during the parent-cohort period, 17 July 2022 to 16 July 2023. Of these, 79 women (23.0%) had documented obstetric haemorrhage and were included in this analysis. The median age was 33 years (IQR: 30–37); median parity was 3 (IQR: 1–4). The median GCS recorded in the source dataset was 14 (IQR: 7–15), systolic blood pressure was 122 mmHg (IQR: 100–146.5), diastolic blood pressure was 80 mmHg (IQR: 60–94), and oxygen saturation was 96% (IQR: 90.5–98.5). Although the dataset labels GCS as an admission measure, its exact measurement time could not be verified. Cohort characteristics, post-admission treatments, and outcomes are summarized in Table 1.

### 3.2. Distribution of Haemorrhage Subtypes and Outcomes

**Figure 1:**
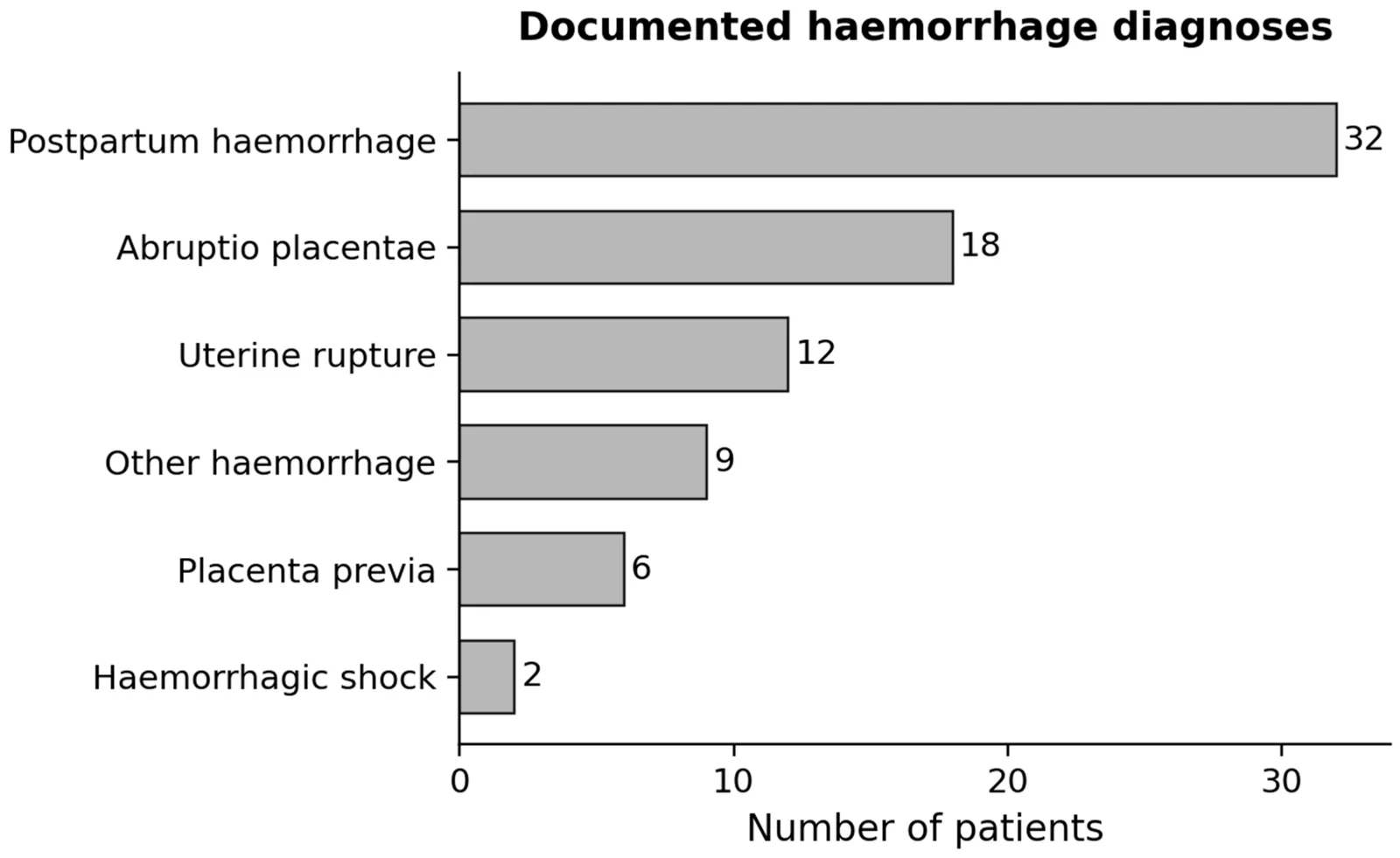
Distribution of documented obstetric haemorrhage diagnoses (N = 79). “Other haemorrhage” denotes documented haemorrhage-related diagnoses not separately classified in the figure; their individual components were unavailable in the aggregate dataset.

Postpartum haemorrhage was the most common documented diagnosis, accounting for 32 cases (40.5%), followed by abruptio placentae (18, 22.8%), uterine rupture (12, 15.2%), other haemorrhage-related diagnoses (9, 11.4%), placenta previa (6, 7.6%), and haemorrhagic shock (2, 2.5%) (Figure 1). The residual “other haemorrhage” category contains diagnoses documented in the records but not separately represented in the analytic categories; the individual diagnoses were not available in the aggregate dataset. Overall, 44 women died in the ICU (55.7%), while 35 (44.3%) survived to ICU discharge. In absolute terms, postpartum haemorrhage accounted for the largest number of deaths (21/32), followed by abruptio placentae (7/18) and uterine rupture (6/12). Case fatality was highest for haemorrhagic shock (2/2, 100%), followed by other haemorrhage-related diagnoses (6/9, 66.7%) and postpartum haemorrhage (21/32, 65.6%). Detailed outcomes by diagnosis are shown in Table 2.

### 3.3. Comparison Between Survivors and Non-Survivors

Compared with survivors, women who died had lower GCS scores as recorded in the source dataset (median 8 vs. 15, *p* < 0.001) and lower diastolic blood pressure (71.5 vs. 86 mmHg, *p* = 0.010). Hospital length of stay was shorter among non-survivors (4 vs. 9 days, *p* < 0.001). Mechanical ventilation was more frequent among non-survivors than survivors (31/44, 70.5% vs. 14/35, 40.0%; *p* = 0.013). Length of stay and ICU treatments describe post-admission course and care intensity; these unadjusted comparisons do not identify baseline predictors or causal effects. Supplemental oxygen was recorded for all 35 survivors and 38 of 44 non-survivors; all six women without a separate supplemental-oxygen record died, and five of the six received mechanical ventilation, through which oxygen may have been delivered. No *p*-value is reported for this comparison, and the difference is not interpreted as evidence that oxygen reduced or increased mortality, because separate oxygen documentation may not reflect total oxygen exposure. No statistically significant differences were observed for age, parity, heart rate, systolic blood pressure, oxygen saturation, respiratory rate, vasopressor support, central venous catheterization, CPAP use, or haemodialysis (Table 3). No patient underwent tracheostomy.

## 4. Discussion

This study describes outcomes among women with documented obstetric haemorrhage admitted to a general ICU in Ghana. Postpartum haemorrhage was the most common diagnosis, ICU mortality was 55.7%, and women who died had lower recorded GCS and diastolic blood pressure in unadjusted comparisons. The GCS variable was labelled as an admission measure in the source dataset, but its exact measurement time could not be verified. These descriptive findings should not be interpreted as independent prognostic effects.

This predominance of postpartum haemorrhage is consistent with the global pattern in which postpartum haemorrhage accounts for the majority of haemorrhage-related maternal deaths [3,6]. Liu et al. reported that postpartum haemorrhage contributes to the majority of haemorrhage-related maternal deaths globally [15], and McLintock and James estimated that more than 80% of obstetric haemorrhage occurs after delivery [6]. Similar patterns have been reported across several African countries, where postpartum haemorrhage remains the leading contributor to severe maternal outcomes [16,17,18,19]. Active management of the third stage of labour, prompt recognition of excessive bleeding, and reliable stocking of uterotonic agents at all levels of the referral chain remain central to preventing this outcome [20,21].

The 55.7% ICU mortality observed here is higher than the 37.5% case-fatality rate reported among 64 women with haemorrhage admitted to the obstetric ICU at Cape Coast Teaching Hospital [13], and higher than the pooled 30.69% mortality across obstetric ICU admissions in the African meta-analysis by Abie et al. [22]. These estimates are not directly comparable: the Cape Coast estimate concerns haemorrhage-specific ICU admissions, whereas the African pooled estimate combines different obstetric diagnoses, hospitals, and ICU models and had substantial between-study heterogeneity. Lee et al. reported a 5.9% haemorrhage case-fatality rate in a broader KATH population, not an ICU-only cohort [7]. The higher mortality in this ICU subgroup may reflect selection of women with more severe illness, but this study did not measure blood loss, referral delay, transfusion timing, or resuscitation sufficiently to attribute the difference to a specific mechanism. Health-system factors such as delayed recognition, transport and referral constraints, blood availability, and access to organ support are plausible contributors and should be assessed directly in future multicentre work.

The lower recorded GCS among non-survivors is consistent with reports among critically ill obstetric patients elsewhere, where impaired neurological status has been associated with poor outcomes and mortality [23,24]. In the present analysis, however, only unadjusted comparisons could be reported because the patient-level dataset required for an admission-only adjusted analysis was not available for analysis. The GCS finding is therefore descriptive and cannot establish an independent association with mortality.

A lower GCS may reflect cerebral hypoperfusion during haemorrhagic shock, but reduced consciousness can also arise from eclampsia, sedative medication, intubation, or other illness. The source dataset labels the value as “admission GCS,” but we could not determine whether it was measured at ICU arrival or was the first GCS recorded during the ICU stay. Timing relative to sedation or intubation was also unavailable. These uncertainties prevent attribution of the observed difference to haemorrhage severity or an independent prognostic effect.

GCS is one component of neurological assessment and should be interpreted alongside bleeding assessment, blood pressure, pulse, perfusion, and response to resuscitation. This study does not validate GCS thresholds for predicting mortality in obstetric haemorrhage. A reduced or declining level of consciousness warrants clinical reassessment, but GCS should not replace standard haemorrhage recognition, resuscitation, or escalation procedures.

Mechanical ventilation was more frequent among women who died, but ventilation and vasopressor support were recorded as ICU treatments rather than baseline exposures. Their use may reflect severe illness and may occur after deterioration; they are therefore described as markers of care intensity, not independent baseline predictors or causes of death. The available analysis did not establish whether ventilation began before ICU transfer or after admission, limiting interpretation of its timing.

Women who died also had lower diastolic blood pressure in the unadjusted comparison. Because no adjusted analysis could be conducted, the study cannot determine whether this difference was independent of other clinical factors. Haemodynamic monitoring remains an essential part of managing obstetric haemorrhage, as early recognition of circulatory compromise can facilitate timely resuscitation.

The findings support attention to actions across the referral pathway: rapid recognition of bleeding, early haemorrhage resuscitation, timely access to blood products and uterotonics, clear escalation criteria, communication between referring facilities and KATH, and prompt access to critical care. The study did not contain enough information to determine which delays occurred, classify the 44 deaths as preventable or unavoidable, or identify their clinical causes. These recommendations are therefore priorities for service review and prospective evaluation, not causal conclusions from this dataset.

### Strengths and Limitations

This study provides diagnosis-specific data from a general mixed ICU, but several limitations affect interpretation. The retrospective, single-centre design limits data completeness and generalizability. Case inclusion relied on diagnoses documented in records; we could not verify a separate KATH operational definition of “severe obstetric haemorrhage”, and estimated blood loss, transfusion thresholds, serial haemoglobin, and shock criteria were unavailable in the analytic data. Although the 79 cases were drawn from the parent-cohort period of 17 July 2022 to 16 July 2023, the exact dates of the first and last haemorrhage-specific admissions could not be confirmed because the ICU admission register was unavailable. This analysis shares its source cohort with a companion paper covering all 344 obstetric ICU admissions; the scope and possible overlap are disclosed in the Methods Section and cover letter. The patient-level dataset required for a prespecified admission-only model was not available for analysis, so results are limited to unadjusted comparisons and no independent prognostic effects can be inferred. The timing of the GCS measurement could not be confirmed as ICU arrival or the first value recorded during the stay, and its relation to sedation or intubation was unavailable. The report does not establish whether abstractors were blinded or whether duplicate abstraction and inter-rater reliability were assessed. Information on pre-referral delay, time to resuscitation, volume and timing of blood transfusion, uterotonic use, and resuscitation status was insufficient or unavailable. Cause-of-death review and preventability classification were unavailable. The small cohort and 44 deaths limit precision and may leave the study underpowered to detect other unadjusted differences.

## 5. Conclusions

Among women admitted to a general mixed ICU with documented obstetric haemorrhage, ICU mortality was high and postpartum haemorrhage was the most common diagnosis. Non-survivors had lower GCS and diastolic blood pressure in unadjusted comparisons, but GCS timing could not be verified and no adjusted prognostic effect can be inferred. The available data do not allow mortality to be classified as preventable or attributed to specific referral or treatment delays. Earlier recognition, prompt resuscitation, reliable referral, timely access to blood and uterotonics, and critical care access are priorities for prospective service evaluation. Multicentre studies with explicit inclusion dates, standardized case criteria, verified admission measurements, treatment timing, and cause-of-death review are needed.

## Figures and Tables

**Table 1:** Cohort characteristics, post-admission ICU treatments, and outcomes among women with documented obstetric haemorrhage (N = 79).

Characteristic	Value
Documented obstetric haemorrhage admissions	79
Demographic and clinical characteristics	
Age, years, median (IQR)	33 (30–37)
Parity, median (IQR)	3 (1–4)
GCS score recorded in source dataset, median (IQR)	14 (7–15)
Heart rate, median (IQR)	99 (83.5–117)
Systolic BP, median (IQR)	122 (100–146.5)
Diastolic BP, median (IQR)	80 (60–94)
SpO2, median (IQR)	96 (90.5–98.5)
Respiratory rate, median (IQR)	23 (18–31)
Referred from another facility	67/79 (84.8%)
Post-admission course and ICU treatments	
Length of hospital stay, days, median (IQR)	7 (4–11)
Mechanical ventilation	45/79 (57.0%)
Vasopressor support	19/79 (24.1%)
CVC	17/79 (21.5%)
CPAP	6/79 (7.6%)
Haemodialysis	2/79 (2.5%)
Tracheostomy	0/79 (0%)
Supplemental oxygen	73/79 (92.4%)
ICU outcomes	
ICU mortality	44/79 (55.7%)
Discharged alive	35/79 (44.3%)

**Table 2:** Distribution and outcomes of documented obstetric haemorrhage by diagnosis. Ventilation and vasopressor support are treatments recorded during ICU care; median hospital length of stay is a post-admission measure reported for all patients in each diagnosis group.

Haemorrhage Subtype	N	Died	Fatality (%)	Ventilation, n (%)	Vasopressors, n (%)	Median LOS (d)
Abruptio placentae	18	7	38.9%	9 (50.0%)	3 (16.7%)	7.0
Haemorrhagic shock	2	2	100.0%	2 (100.0%)	1 (50.0%)	2.5
Other haemorrhage	9	6	66.7%	5 (55.6%)	2 (22.2%)	4.0
Placenta previa	6	2	33.3%	3 (50.0%)	1 (16.7%)	20.0
Postpartum haemorrhage	32	21	65.6%	20 (62.5%)	8 (25.0%)	7.0
Uterine rupture	12	6	50.0%	6 (50.0%)	4 (33.3%)	5.5

**Table 3:** Unadjusted comparison of demographic and clinical characteristics, post-admission treatments, and course between ICU survivors and non-survivors.

Characteristic	Died (n = 44)	Survived (n = 35)	*p*-Value
Demographic and clinical characteristics			
Age	32 (29.8–35)	33 (30.5–38)	0.112
Parity	2.5 (1–4)	3 (1–4)	0.924
GCS score recorded in source dataset	8 (5–15)	15 (14.5–15)	<0.001
Heart rate	100.5 (79.8–129)	99 (89–112.5)	0.921
Systolic BP	121 (96.2–140.2)	126 (112–152.5)	0.066
Diastolic BP	71.5 (52–88)	86 (73–98)	0.010
SpO2	96 (88.8–98.2)	96 (92.5–98.5)	0.673
Respiratory rate	23 (17.8–31)	23 (19.5–30.5)	0.556
Post-admission course and ICU treatments			
Length of stay (days)	4 (2–7)	9 (7–13)	<0.001
Mechanical ventilation	31 (70.5)	14 (40.0)	0.013
Vasopressor support	14 (31.8)	5 (14.3)	0.122
CVC	10 (22.7)	7 (20.0)	0.986
CPAP	2 (4.5)	4 (11.4)	0.398
Haemodialysis	1 (2.3)	1 (2.9)	1.000
Tracheostomy	0 (0.0)	0 (0.0)	NE
Supplemental oxygen	38 (86.4)	35 (100.0)	-

Continuous variables are presented as median (IQR) and categorical variables as n (%). GCS is labelled “admission GCS” in the source dataset, but its exact measurement time could not be verified. ICU treatments were recorded during the ICU stay and are post-admission variables, not baseline risk factors. NE, not estimable: no patient in either group underwent tracheostomy. Supplemental oxygen is presented descriptively; no *p*-value is reported because a separate oxygen record may not capture oxygen delivered through mechanical ventilation or reflect clinical indication.

## Data Availability

The aggregate data supporting the findings are presented in the manuscript. The patient-level dataset required for additional adjusted analyses was not available for analysis. Requests for access to underlying records may be directed to the corresponding author and would be subject to KATH institutional permissions.
